# How to Optimize Healthy Volunteer Screening and Enrolment in Clinical Trials: A Qualitative and Quantitative Approach to Evaluate Processes and Healthy Volunteers' Perceptions

**DOI:** 10.1002/cpt.70462

**Published:** 2026-09-09

**Authors:** Evelyn Krohmer, Manuela König, Cathrin J. Vogt, Marina Streckebein, Sybille Baumann, Max Blumenstock, Ruwen Böhm, Fabian Müller, Nadja Struß, Viktoria S. Wurmbach, Antje Blank

**Affiliations:** ^1^ Internal Medicine IX, Department of Clinical Pharmacology and Pharmacoepidemiology Medical Faculty Heidelberg University/Heidelberg University Hospital Heidelberg Germany; ^2^ CRS hVIVO Mannheim Germany; ^3^ CRS Berlin Germany; ^4^ Institute of Medical Informatics Heidelberg University Heidelberg Germany; ^5^ Socratec Erfurt Germany; ^6^ Boehringer Ingelheim Pharma GmbH & Co. KG Biberach an der Riß Germany; ^7^ Institute of Experimental and Clinical Pharmacology and Toxicology Friedrich‐Alexander‐Universität Erlangen‐Nürnberg Erlangen Germany

## Abstract

Healthy volunteers (HVs) are essential for phase I trials, yet their recruitment and screening remain resource‐intensive and ethically complex. High screening failure (SF) rates highlight the need to evaluate and understand all aspects of the enrolment processes. We analyzed SF patterns in a large German HV cohort and explored participants' motivations, risk perceptions, and expectations regarding trial participation and screening procedure. We retrospectively evaluated screening results from phase I trials (2012–2022) at Heidelberg University Hospital. Additionally, we performed a prospective mixed‐methods assessment with semi‐structured interviews and a questionnaire evaluating recruitment, informed consent (IC), motivation, and decision making, distributed to experienced volunteers of four trial sites (October 2024–August 2025). Across 30 phase I trials, 900 volunteers were screened with a SF rate of 31.9% (*n* = 287), mainly due to laboratory abnormalities, medical history of concern, and organizational issues. Results from five semi‐structured interviews and the questionnaire survey (*n* = 196) showed an overall acceptable satisfaction with the IC process, but identified areas of improvement in weighting of topics and risk perception. Although financial compensation was voted as the most important motivation, the decision process is multifactorial and an interplay of compensation, organizational aspects, and expected risks. We identified trial characteristics that are modifiable to facilitate interest in trial participation and provide data‐driven recommendations to improve the ethical, efficient, and operational conduct of phase I trials. Furthermore, the results underline the relevance of addressing public perceptions and misconceptions surrounding clinical trials to improve willingness to engage in early‐phase research.

**Study Highlights**

**WHAT IS THE CURRENT KNOWLEDGE ON THE TOPIC?**

Screening failure (SF) rates in phase I clinical trials with healthy volunteers (HVs) are high and contribute substantially to delays, costs, and inefficient use of resources in drug development. Financial compensation is the most important motivator for HVs to participate in clinical trials.

**WHAT QUESTION DID THIS STUDY ADDRESS?**

This study examined why HVs participate in phase I clinical trials and why screening frequently fails, combining a retrospective analysis of screening outcomes with qualitative and quantitative assessments of volunteer motivation, perception, and preferences.

**WHAT DOES THIS STUDY ADD TO OUR KNOWLEDGE?**

The SF rates observed at a German academic phase I unit were lower than those reported in several international cohorts. Key drivers of SF were abnormal laboratory values and medical history. SF reasons are strongly dependent on population characteristics, particularly age distribution and chosen eligibility criteria. While financial compensation is a major motivator for HVs, participation decisions also incorporate perceived burden, risk, and organizational factors. The study further reveals persistent misconceptions among volunteers regarding trial participation, risk, and financial compensation, as well as modifiable organizational factors influencing enrolment.

**HOW MIGHT THIS CHANGE CLINICAL PHARMACOLOGY OR TRANSLATIONAL SCIENCE?**

Developing data‐driven, risk‐adapted eligibility criteria and optimizing screening workflows may reduce screening failures and resource burden in phase I trials. A participant‐centered approach to recruitment, informed consent, and study design—combined with improved public communication addressing misconceptions—may enhance ethical conduct, recruitment efficiency, and the sustainability of translational clinical research.

Clinical trials involving healthy volunteers (HVs) are essential to translational clinical research and drug innovation. Especially phase I trials commonly require the enrolment of HVs for obtaining initial data on investigational drugs under conditions of normal human physiology, establishing the basis for their safe development in patients. Their enrolment is allowed based on pertinent laws^1^ and guidelines^2^ and is unavoidably associated with potential risks that must be carefully assessed and managed to ensure participant safety.

From a sponsor's perspective, the enrolment process, comprising recruitment, informed consent (IC), and medical screening, is time‐consuming and costly. Deficiencies in any of these parts can significantly impact the ethical integrity and operational success of a trial. Each part has its own challenges and is embedded in a social, legal, and cultural context that differs across regions.

Recruitment delays remain a challenge, potentially leading to insufficient sample sizes, underpowered results, or costly trial extensions.^3^, ^4^ Successful recruitment depends on understanding HVs motivations. Financial compensation is the primary motivator for most HVs, although other factors such as time commitment, scientific interest, altruism, and social aspects also influence their decision making.^5^ Concerns persist regarding potential exploitation of low‐income volunteers^4^ which is also demonstrated in societal reflections of study participation facilitating ongoing prejudices. In contrast to that, while financial compensation ranks highly in trade‐off scenarios, perceived risks often outweigh the financial rewards.^6^ Recruitment strategies should aim to avoid selection bias of HV populations that do not adequately represent the composition of the society and may limit the external validity of the trial's findings.^7^

Prior to screening, properly conducted IC is critical for ethical trial conduct, ensuring participants' autonomy and their understanding of key aspects, such as the trial procedures, risks, benefits, and data protection. Poor comprehension is an ethical issue and may lead to non‐compliance, withdrawal, or adverse events, ultimately compromising study success, validity, and safety. Despite its importance, phase I participants show limited understanding of the study's purpose and the drugs administered, while demonstrating good knowledge of administration route or financial compensation.^8^ Although the involvement of laypersons during the planning phase of clinical trial has already begun, data on expectations and wishes of HVs for the IC remain scarce.

Following recruitment and IC, extensive screening examinations are conducted to determine participant eligibility. Guidance on eligibility assessment was provided by Breithaupt‐Groegler et al.^9^ These criteria are enforced by German authorities and are therefore present in most phase I trial protocols in Germany. Screening failure (SF) rates reported from various regions are high, ranging from 39.8% in a UK vaccine trial^10^ to 42% in France,^11^ 53% in India,^12^ and 72% in China^13^ for HV phase I trials. Frequent causes of failure are inappropriate medical history,^11^ abnormal laboratory results,^11^, ^12^, ^13^ and vital sign abnormalities, especially among senior or overweight volunteers.^11^, ^14^

The aim of this study was to qualitatively and quantitatively analyze the enrolment of HV in phase I trials, identify areas for improvement, and provide recommendations to support ethically sound, fast, and efficient conduct of translational trials. We therefore combined a retrospective analysis of SF reasons in a large German HV population with a prospective evaluation of motivation, perception, and preferences of experienced HVs.

## MATERIAL AND METHODS

This study comprises three parts: a retrospective analysis of screening failures in HV trials conducted over 10 years at a German academic trial site, followed by a prospective qualitative analysis and a prospective quantitative analysis, both investigating preferences and perception of clinical trials from the perspective of experienced HVs in Germany. It was approved by the responsible ethics committee (Ethics Committee of the Medical Faculty Heidelberg label: S‐224/2023) and registered (DRKS00039582).

### Retrospective analysis

We retrospectively evaluated all HV screening visits conducted between 01/2012 and 01/2022 in 30 phase I trials at the ISO‐certified Pharmacological Early Clinical Trial Unit of the Heidelberg University Hospital. Participants who had provided written IC and undergone at least one screening assessment were included. Screening assessments typically included evaluation of medical history, prior and concomitant drug use, current symptoms, physical examination, vital signs, laboratory testing (blood and urine), electrocardiogram (ECG), and protocol‐specific assessments.

As SF reasons were documented heterogeneously across trials, source data were retrieved from trial archives and entered into the IMI‐EDC electronic data capture system.^15^ Eligibility criteria changed over the 10‐year study period, and screening failures were classified according to the criteria that were defined in the respective trial. Findings leading to SF were either defined by protocol‐specified cutoff values or, in the absence of such thresholds, considered a clinically significant abnormality by the investigator at that time. Descriptive analysis was performed by two independent reviewers with RStudio version 4.3.1 (R Core Team, 2023) and compared for consistency.

### Semi‐structured interviews

Five semi‐structured interviews were conducted with randomly selected experienced HVs, who had participated in at least one previous clinical trial and were actively participating in a trial at the time of the interviews. After written IC, participants were interviewed using a guide developed from a literature review and comprising four main open‐ended questions, addressing experience with trial participation, their decision process, recruitment, and IC. Interviews were transcribed and qualitatively analyzed by two independent coders with MAXQDA (VERBI—Software. Consult. Sozialforschung. GmbH, Berlin, Germany). The code list was generated inductively from the material and summarized into 10 different main categories (**Table** **S1**) to capture the perception and expectations of HV of clinical trials. The frequency of coded sections was analyzed descriptively.

### Questionnaire

Based on the above‐mentioned literature research and the interview results, a structured questionnaire was developed in a stepwise process at the clinical trial site and examined for clarity, comprehensibility, and unambiguity according to the question assessment criteria based on the principles of Faulbaum et al.^16^ The pre‐final survey was piloted in four individual interviews with cognitive probing techniques^16^ and adjusted by the research group where needed. The final questionnaire used 3‐ to 5‐point Likert scales, closed single‐ and multiple‐choice items, and free‐text questions, covering five domains (**Table** **S2**).

Eligible participants were HVs who had previously attended a screening visit within the preceding 24 months. Four German phase I units (one academic and three commercial) distributed an anonymized LimeSurvey (LimeSurvey GmbH, Hamburg, Germany) link to HVs registered as prior participants in their databases. Due to the anonymization and protection of trial staff, site‐specific analyses were not possible. Data were collected between October 2024 and August 2025.

Incomplete questionnaires were excluded. Descriptive analysis included absolute and relative frequency^17^ and Spearman rank correlation^18^ with a coefficient of > 0.3 considered relevant. Free‐text answers were coded with MAXQDA by two independent coders and analyzed descriptively.

## RESULTS

### Retrospective analysis of screening failure reasons

In 10 years, exactly 900 volunteers participated in screening visits in 30 phase I trials (26 investigator‐initiated, 4 sponsor‐initiated). Study types included drug–drug interactions (*n* = 13), pharmacokinetics (*n* = 12), first‐in‐human studies (*n* = 3), bioequivalence (*n* = 1), and pharmacodynamics (*n* = 1) (**Table** **S3**).

Participants had a median age of 26 years (y), were evenly distributed by sex, predominantly Caucasian (79%), and 59% had previous trial experience (**Table** **S4**). Population variables stratified for screening outcome are shown in **Table** **1**.

**Table 1:** Socio‐demographic characteristics of 900 screened healthy volunteers stratified for screening outcome

Characteristics	Category	Screening failure (*n* = 287)	Screening success (*n* = 613)
Age [y]^a^		25 (18–63)	27 (18–63)
BMI [kg/m^2^]^b^		24 (±4.6)	24 (±3.3)
Ethnic background	White/Caucasian	207 (72%)	504 (82%)
	Not given/Other	62 (22%)	79 (13%)
	Asian	8 (2.8%)	10 (1.6%)
	Hispanic or Latino	6 (2.1%)	6 (1.0%)
	More than one ethnicity	3 (1.0%)	14 (2.3%)
	Black or African American	1 (0.3%)	0 (0%)
Occupation	Unknown	157 (55%)	274 (45%)
	Student	101 (35%)	239 (39%)
	Employed	26 (9.1%)	93 (15%)
	Unemployed	3 (1.0%)	7 (1.1%)
Prior trial participation	No participation in any trials	144 (50%)	209 (34%)
	Previous participation in trials	117 (41%)	390 (64%)
	Unknown	26 (9%)	14 (2.3%)
Prior participation in a clinical trial^c^	Non‐clinical trial	29 (25%)	54 (14%)
	Clinical trial	35 (30%)	199 (51%)
	No further information given	53 (45%)	137 (35%)
Sex	Female	150 (52%)	320 (52%)
	Male	134 (47%)	292 (48%)
	Unknown	3 (1.0)	1 (0.2%)

Values are reported as total (percent) unless declared otherwise. Missing data may be the case, when the screening process was prematurely ended due to identification of exclusion criteria. For four participants, source data sheets were not available. Due to rounding, percentages may not add up to 100%. BMI, body mass index.

^a^ Age is reported as median (range).

^b^ BMI is reported as mean (± standard deviation).

^c^ This assessment includes only HVs who reported to have previously participated in trials.

The overall SF rate of all analyzed studies was 31.9% (287 of 900 participants). As some volunteers fulfilled multiple exclusion criteria, 330 SF reasons were identified. The most common cause for SF was abnormal laboratory values in 115 cases (35%), followed by medical history (15%) as shown in **Table** **2**.

**Table 2:** Absolute and relative distribution of screening failure reasons

Screening failure reason	Total number of SF reasons [*n*] (Total number of findings [*n*]^a^)	Relative frequency [%]
Abnormal Laboratory values (incl. blood and urine test)	115 (181)	34.8
Abnormal medical history	49 (76)	14.8
Organizational reasons	27	8.2
Withdrawal of IC/No IC given	24	7.3
Abnormal vital signs	23	7.0
Abnormal ECG evaluation	22	6.7
Study specific reasons	20	6.1
Prohibited medication or drugs	18	5.5
Other	12	3.6
Abnormal physical examination	5	1.5
Acute illness	5	1.5
Poor vein status	4	1.2
Smoking	3	0.9
Suspected non‐compliance	3	0.9
Total	330	100.0

Participants could have more than one screen failure reason; therefore, the total number of screen failure reasons exceeds the total number of excluded participants (*n* = 287). ECG, electrocardiogram; IC, informed consent.

^a^ For some categories, multiple findings could be reported for a single participant.

**Figure 1:**
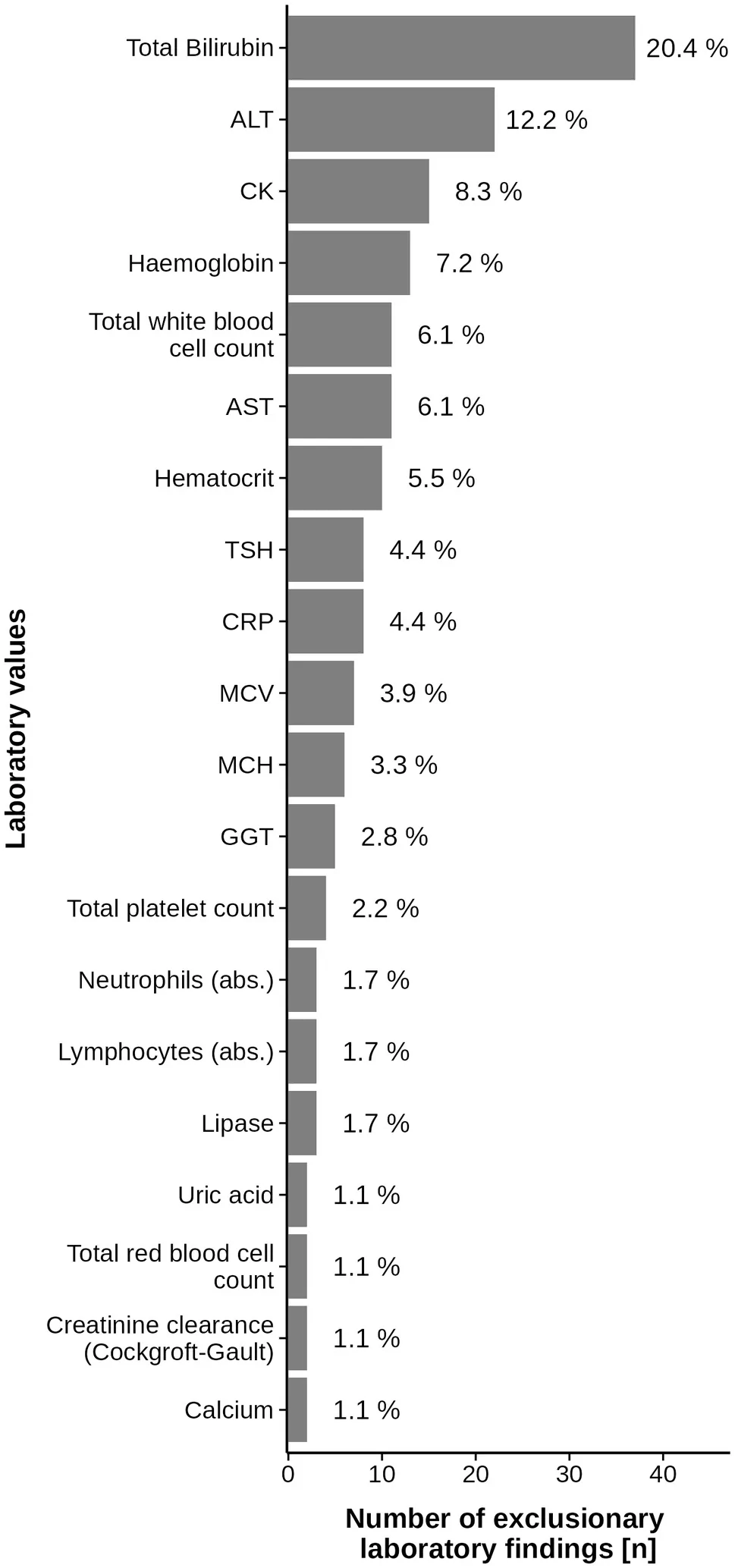
Distribution of laboratory abnormalities leading to screening failure. Abnormal findings were defined either by exceeding a protocol‐specified cutoff value or by being assessed as clinically significant by the investigator at the time of the respective trial. ALT: alanine aminotransferase; AST: aspartate aminotransferase; CK: creatine kinase; CRP: C‐reactive protein; GGT: gamma‐glutamyl transferase; MCH: mean corpuscular hemoglobin; MCV: mean corpuscular volume; TSH: Thyroid stimulating hormone. Only findings with a relative frequency > 1% are shown. Percentages are based on the total number of abnormal laboratory findings (*n* = 181), not on the number of participants, as individual participants could contribute multiple abnormal laboratory findings leading to exclusion. Percentage values are displayed to the right of each bar.

From 181 total number of abnormal laboratory findings, elevated total bilirubin was the most frequent in 37 cases (20.4%), followed by alanine aminotransferase (*n* = 22, 12.2%), creatine kinase (*n* = 15, 8.3%), hemoglobin (*n* = 13, 7.2%), and aspartate aminotransferase (*n* = 11, 6.1%) as shown in **Figure** **1**.

**Figure 2:**
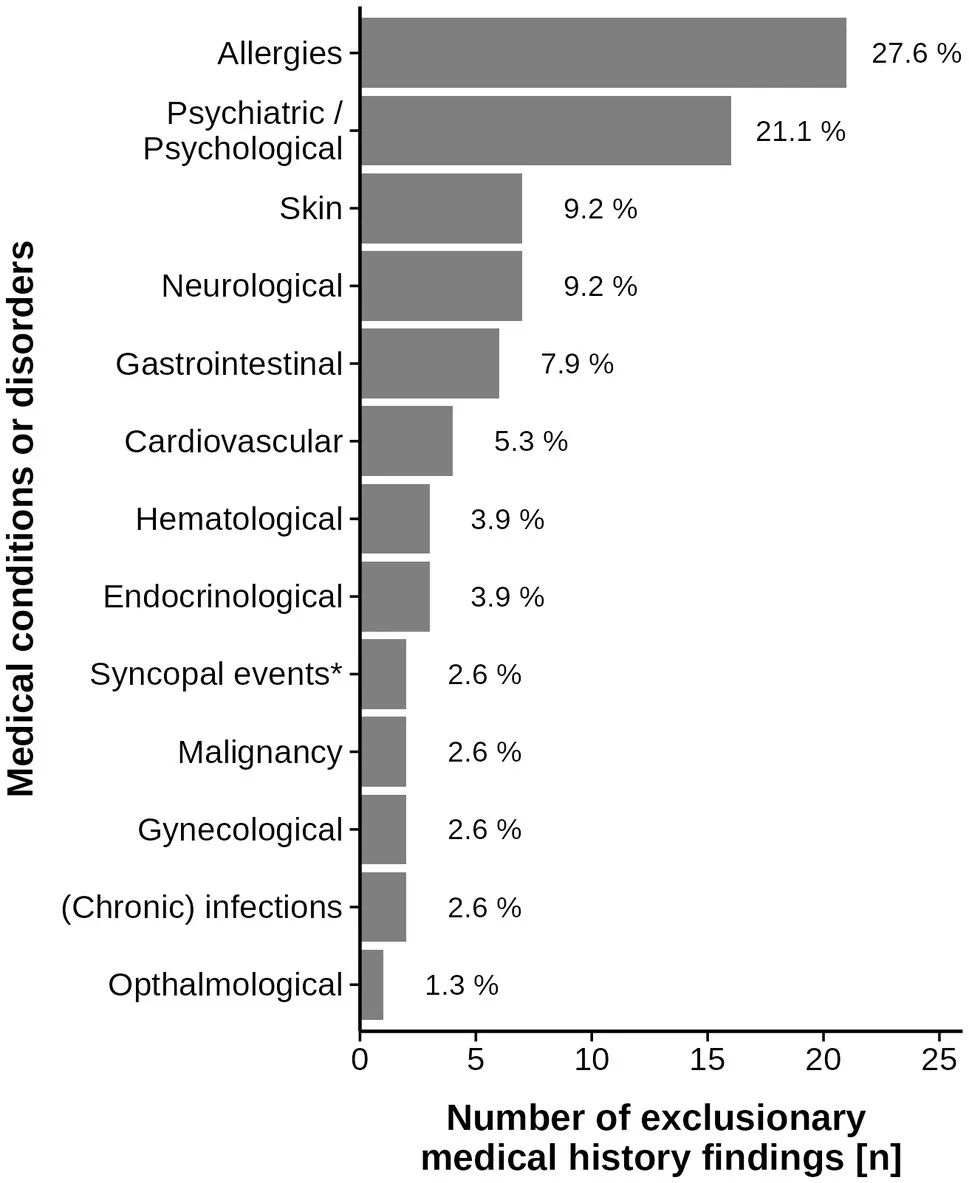
Distribution of abnormal medical history leading to screening failure. *includes Fainting, Dizziness, Unconsciousness, Arrhythmia, or Bradycardia. Percentages are based on the total number of abnormal medical history findings (*n* = 76), not on the number of participants, as individual participants could contribute multiple abnormal findings leading to exclusion. Percentage values are displayed to the right of each bar.

Medical history as second leading cause with a total number of 76 abnormal medical history findings in 49 cases, most often involved clinically relevant allergies (*n* = 21, 27.6%), followed by psychiatric or psychological conditions (*n* = 16, 21.1%) as shown in **Figure** **2**.

Participants with abnormal parameters could be re‐tested if the condition needed clarification or was thought to be the result of a transient and reversible condition. Re‐testing occurred in 104 cases mainly for repetition of blood biochemistry (73%) after which 61 (59%) could be included (**Table** **S5**).

Re‐screening may be necessary if participants could not be included in the protocol‐specific timeframe (usually within 30 days after the screening visit) and was conducted for 24 participants, with the most common cause being organizational reasons (*n* = 9; 38%), after which 19 (79%) were included (**Table** **S6**).

### Qualitative analysis of experienced healthy volunteers on their perception of clinical trials

HVs reported both positive and negative experiences with trial participation. Many positive experiences included organizational aspects like study duration, scheduling, and communication with the study team. Friendliness and professionalism were important for the participants' sense of comfort and safety.

Negative experiences were primarily associated with study design and procedures; participants described discomfort related to specific interventions, adverse drug reactions, time expenditure, study‐related restrictions, and aspects of the study environment. In particular, limited privacy and lack of comfort during on‐site stays was a recurring theme, as pointed out by this participant:

“[…] you have nowhere to retreat to, not in your room and not here, so you don't really have any opportunity to just be yourself. […] I don't know who chose my meals, but I always got the vegetarian menu and it got worse by the day.”

**Figure 3:**
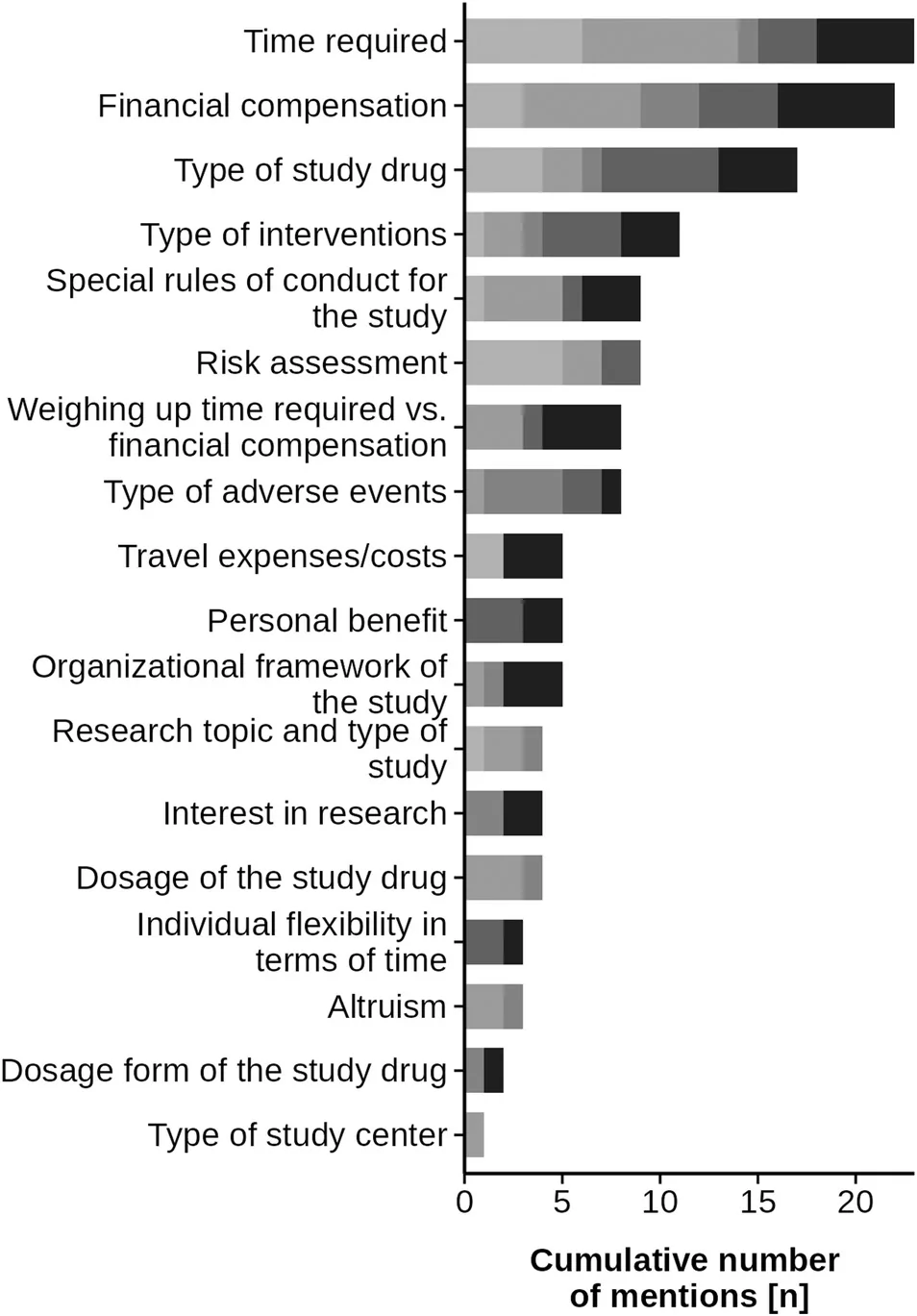
Factors influencing the decision for or against study participation mentioned during the interviews. Bars represent the cumulative number of mentions across all five interviews. Different shades of gray indicate the number of mentions contributed by each individual participant. Repeated mentions of the same factor within a single interview were counted as one mention each.

Three central factors for decision making emerged from the interviews: financial compensation, time and organizational requirements, and perceived risk. Participants described weighting these factors against one another, often relying on previous trial experience as guidance. Other commonly cited motivators, such as altruism, interest in research, or the specific research topic were mentioned less frequently (**Figure** **3**).

Perceived risk appeared as a particularly complex factor, shaped by both objective study characteristics and subjective perceptions and beliefs. Participants considered the type of study drug, planned interventions, expected adverse events, and mode of administration as well as concerns about unknown or long‐term side effects, general pharmaceutical skepticism, or negative societal prejudices, as highlighted by this participant:

“Yes, I think there is this stigma […] that if you participate in [trials] now, […] that you have to live with the risks […] and perhaps diseases will develop or result from it. […] all the portrayals I've ever seen in movies, television, and the media have never been positive. […] it wasn't a relatively boring, controlled process, but rather that you're somehow like Deadpool or someone who is so ravaged by poverty that you're forced to destroy yourself[.] […] that somehow reinforces this stigma a little bit.”

This was reflected in negative reactions from participants' social environments, suggesting that societal narratives may influence attitudes toward clinical trial participation.

Consequently, clear communication during the IC process becomes particularly important. Overall, HVs evaluated past IC discussions positively, reported good comprehension of the study, and felt well‐informed. However, the excessive duration and repetitive nature of the discussion were mentioned as burdens.

In summary, motivation to participate in clinical trials is multifactorial and shaped by both modifiable and non‐modifiable factors. While aspects such as trial design, on‐site conditions, and participant experience can be optimized by sponsors and trial sites, others, including the investigational product and its associated risk profile, remain largely fixed.

### Quantitative analysis of healthy volunteer motivation, decision making and preferences

A total of 196 completed questionnaires were analyzed. Participants were evenly distributed across the age groups; half of the participants were female (52%), reported to be in a relationship (52%), and had a university degree (52%), and income was equally distributed across the monthly income groups up to 5000 € as shown in **Table** **3**.

**Table 3:** Socio‐demographic characteristics of 196 questionnaire participants

Characteristic	Category	Total [*n*]	Percentage [%]
Age [y]	18–24	18	9
	25–30	31	16
	31–40	56	29
	41–50	33	17
	Older than 50	58	30
Sex	Male	94	48
	Female	102	52
Education	High school diploma	27	14
	Vocational training	36	18
	University degree	101	52
	Middle school diploma	26	13
	PhD	6	3
Marital	In a relationship	101	52
	Single	95	48
Monthly income [€]	Less than 1000	26	13
	1000–1499	38	19
	1500–1999	26	13
	2000–3499	54	28
	3500–4999	34	17
	5000 or more	18	9
Number of study participations [*n*]	0^a^	7	4
	1	46	23
	2–4	54	28
	5–9	51	26
	10 or more	38	19

^a^ Participants completed full screening, but were not included in a trial (screening failures).

Recruitment commonly occurred via active online search (49%) and contact through study team (42%), both methods positively rated (“somewhat promising” or “promising”) in the majority of cases (76% and 74%). Social media was successful in 18% of cases, while positively rated in 63% of cases. Newspaper as a recruitment method was reported to play a minor role (18%).

Participants' satisfaction with their last IC, which was within the preceding 12 months in the majority of cases (83%), was high. The participants agreed or somewhat agreed that it had good comprehensibility (94%), adequate time expenditure (88%), adequate weighting of the different aspects (94%), and left no open questions (95%). The information rated most important were the time schedule of the study (95%), financial compensation (92%), and expected adverse events (89%), while study personnel, the possibility to look up study results, and the handling of the biomaterial were rated lowest (**Figure** **S1**). No significant correlations were observed between demographic factors and information priorities.

**Figure 4:**
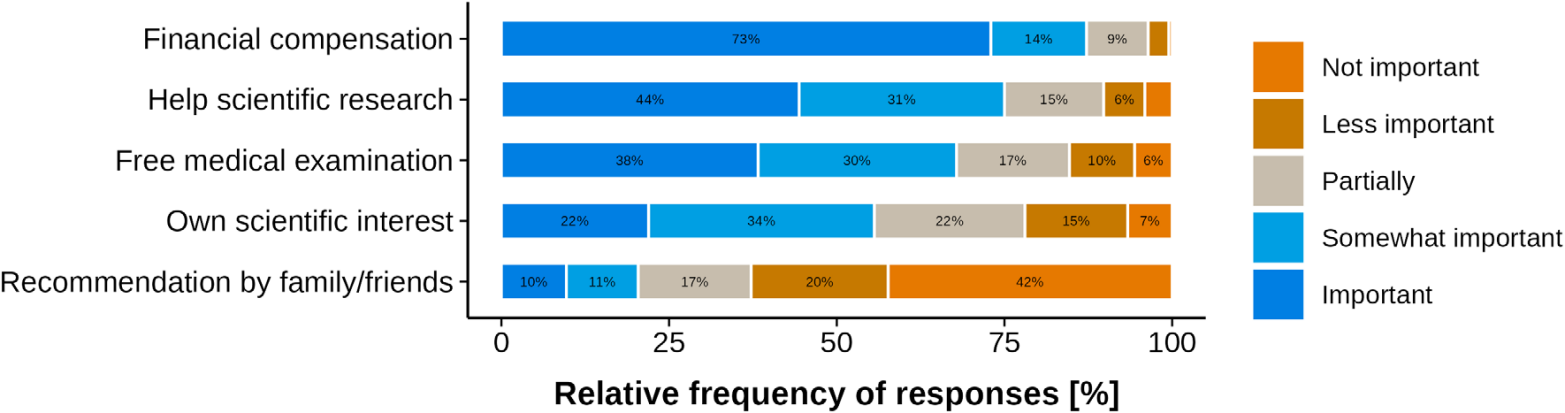
Rated reasons for trial participation. Percentage values >5% are displayed in the corresponding bar.

Financial compensation was rated most important as a reason for trial participation (87% voted important or somewhat important), followed by helping scientific research (75%), getting a free medical examination (68%), and own scientific interest (56%) as shown in **Figure** **4**.

There was no significant correlation between the rating of different reasons and the population characteristics (**Figure** **S2**). Further reasons mentioned in the free‐text answer fields were enjoying “me‐time” during a study day (*n* = 6) and contact to new people (*n* = 2).

The questionnaire responses reflected the interview findings, identifying financial compensation, time commitment, and perceived risk as primary decision factors as well as the proportion between financial compensation to time (90% rated “important” or “somewhat important”) and risk (81%). There was no significant correlation between population characteristics and the importance rating of different aspects for study participation (**Figure** **S3**).

The willingness to participate depended strongly on study type and intervention invasiveness, while the type of drug administration was less influential. Although higher‐risk designs, identified as problematic in the literature,^19^ were viewed more critically, more than half of the volunteers would still participate in trials involving anticancer drugs (80%), first‐in‐human trials (62%), new vaccinations (61%), or immune‐modulating drugs (55%). Drugs affecting the brain were least accepted (42%), which is also reflected in the free‐text responses, where trials involving psychotropic drugs were most frequently rejected (*n* = 23), and adverse events related to the nervous system were cited as the most concerning (*n* = 63). Willingness to undergo invasive procedures was lower, with 61% reluctant to lumbar and 59% to bone marrow puncture, while less invasive but uncomfortable procedures were widely accepted. The route of drug administration had minor impact; injections were slightly less accepted (91% would participate or rather participate) than oral intake (100%). Lifestyle restrictions were generally accepted for specific food (99%), nicotine (99%), and alcohol (98%), but less so for coffee (87%) and sports (71%).

Following the interview results, we wanted to know what HV think about the public perception of trial participation and regulation. Although almost all participants reported that they would participate or probably participate in future trials (98%) and would recommend trial participation to others (91% probably yes or definitely), they were more reluctant to disclose their own trial participation. Only 41% would “tell many people,” while the majority would “only tell selected people” (58%). This discrepancy suggests that concerns about the societal perception of clinical trial participation persist. Even among experienced study participants, misconceptions regarding trial regulation were also observed. When asked about factors influencing financial compensation, 73% rated medical risk and 60% rated the study sponsor to have a “very high” or “rather high” impact on compensation, although these factors are not intended to determine compensation according to ethical guidelines.

## DISCUSSION

By integrating retrospective screening data with qualitative and quantitative perspectives of experienced HVs, this study provides a comprehensive analysis of recruitment and enrolment in phase I trials.

### Who is a healthy volunteer?

Across 30 phase I trials conducted over 10 years, we observed a SF rate of 31.9%. Although lower than several published reports,^10^, ^11^, ^12^, ^13^ it remains relevant and confirms that HV screening is resource‐intensive. Differences in reported SF reasons likely reflect variability in study populations, trial designs, and the strictness of eligibility criteria but may also be influenced by procedural differences in screening workflows.

In our analysis, abnormal laboratory results and medical history were the most frequent SF reasons, whereas other studies reported higher rates of abnormal physical examination, vital signs, and ECG findings.^11^, ^13^ Similarly, a large retrospective analysis^14^ identified cardiovascular, metabolic, renal, and hepatic parameters as major contributors to predicted SF reasons, with clear age‐dependent effects. Younger HV may be more often excluded due to low heart rate, while older participants have higher exclusion rates related to blood pressure, QTc, and renal function. As only 7.4% of our population was older than 50 years, this likely explains the observed differences.

Collectively, these findings support our position that data‐driven and risk‐based refinement of eligibility criteria for HVs is necessary. Phase I trials differ widely in their investigational medicinal products (IMP), designs, and expected adverse events, yet this variability is not reflected by current eligibility criteria. For example, age cutoffs above 45 years, as frequently requested by ethics committees or authorities, may not be necessary in low‐risk trials, such as drug–drug interaction studies with marketed products. Similarly, laboratory thresholds could be adapted to the expected toxicity, and the clinical relevance of single, isolated abnormal laboratory results in HVs needs further discussion. Especially, Gilbert's syndrome is an important consideration when defining bilirubin‐based eligibility criteria. Although clinically benign, elevated bilirubin values observed during trial conduct may raise safety concerns and potentially affect study continuation. Evaluation of bilirubin values should therefore be supported by longitudinal bilirubin measurements and/or an extended laboratory assessment to exclude alternative causes of hyperbilirubinemia. Available data indicate that bilirubin concentrations in individuals with Gilbert's syndrome frequently exceed thresholds commonly applied in clinical trials. Total bilirubin concentrations of up to 3.27 ± 1.29 mg/dL following caloric restriction^20^ and mean concentrations of 3.33 mg/dL in carriers of UGT1A1 variants^21^ have been reported, compared with < 1 mg/dL in controls. Nevertheless, bilirubin cutoffs applied in our studies, even with suspected Gilbert's syndrome, typically range from 1.5 × ULN to 3 × ULN, thereby excluding a substantial proportion of otherwise healthy individuals with this condition. Further discussion is therefore warranted to establish evidence‐based bilirubin thresholds that adequately account for the physiological variability while maintaining participant safety. While laboratory criteria are usually defined with cutoff values, medical history criteria are often vaguely defined as “per investigator's discretion.” This is especially challenging for psychiatric conditions in light of the rising numbers of ambulatory psychiatric treatment^22^ and the variability in interpretation may result in differences in participant eligibility assessments across trial sites. Clearer guidelines in these areas may help to avoid inconsistent interpretation and possibly unnecessary exclusion of otherwise suitable volunteers without compromising their safety. Combining additional approaches, such as precise pre‐study information, which may also include a self‐assessment questionnaire on eligibility, early laboratory pre‐screenings (with a separate, general IC), or sequential testing of screening parameters may help reduce costs and time expenses. Overall, our data suggest that current eligibility criteria for HVs may benefit from systematic re‐evaluation.

### Why do healthy volunteers participate?

HVs decisions to participate in phase I trials appear to be primarily driven by a trade‐off between financial compensation and perceived study‐related burden and risk. This is not really surprising and was consistently confirmed by the qualitative interviews and the questionnaire responses. However, it is helpful to explore the details of these views.

Non‐modifiable trial characteristics, such as study type, indication, dosage, and anticipated adverse events are central to risk assessment and strongly influence participation decisions. Higher perceived risk and greater invasiveness reduce the willingness to participate. This is consistent with preceding survey data, showing lower acceptance of trials perceived as “high‐risk” due to their IMP, such as first‐in‐human trials,^23^ or of trials with relevant invasiveness and discomfort of the planned procedures.^6^, ^19^, ^24^ These observations highlight the importance of minimizing procedural burden where possible, which could be achieved by, for example, less painful assessments, reduced sampling frequency, or supportive methods like ultrasound‐assisted venepuncture.

Although financial compensation is the primary motivation, participation decisions were multi‐dimensional. More than half of the participants rated additional factors, such as helping scientific research or a free medical examination as important. This is in line with previous reports and highlights the relevance of a differentiated view on HV motivations.^5^ These may also vary in an age‐dependent manner, as younger volunteers more often reported financial compensation as their primary motive, while older volunteers report greater importance of altruistic or health‐related motivations.^25^ Data on correlation between participant characteristics and participation reasons were mixed^3^, ^25^, ^26^; no significant correlation was seen in our population.

In contrast to non‐modifiable factors, several aspects of trial participation are modifiable and largely organizational or contextual: recruitment strategies, the complexity of the trial design, on‐site conditions (e.g., comfort or offered food), management of appointments, and communication with the study team. Previous studies have identified inconvenient time requirements, pretrial restrictions or lack of recruitment information as part of key barriers^26^, ^27^ and suggested facilitation of convenience and comfort at the trial site as an important target of optimization strategies.^25^ In our analysis, experienced HVs reported active online searches and direct communication with study teams as most successful recruitment pathways. Therefore, professionalized web presence and structured HV databases are important for recruitment optimization.

Interestingly, our findings indicate a remarkably high willingness of HVs to participate in future clinical trials, with almost all respondents expressing at least a probable intention to re‐enroll and a large majority willing to recommend participation to others. Data from the general population^28^ show substantially lower (49.9%) willingness to participate, which is likely reflected by fundamental differences in participant context, where patient willingness is more strongly driven by therapeutic expectations, particularly the prospect of improving one's own health or accessing innovative treatments. Still, the same barriers such as safety concerns, lack of trust, and insufficient knowledge appear to play a prominent role, contributing to uncertainty and lower participation rates.

Overall, while certain factors of perceived risk cannot be modified, participation rates may be improved through optimization of modifiable factors. One important contextual influence is the persistent negative public image of clinical trial participation, as outlined by the discrepancy of volunteers' high willingness to participate in future trials and reluctance to openly share their involvement. Similar patterns of negative attitudes toward trial participation and disapproval by peers have been reported previously.^27^, ^29^ Combined with prejudices about HVs' motivation (“they only do it for the money”), misunderstanding of ethical standards—such as the widespread belief that the medical risk is financially compensated, even among experienced HVs—and fear of serious complications,^29^ these factors contribute to the persistent negative image. Structured and proactive public communication may therefore contribute to a more balanced societal understanding of phase I research and facilitate willingness of study participation for new HVs.

### Does informed consent meet the volunteers' expectations?

Overall satisfaction with the IC process was high. However, participants suggested redistributing time toward information they considered most relevant (e.g., adverse events and procedural burden), implying that IC optimization should prioritize clarity and relevance rather than mere extensiveness. This may be achieved through involvement of volunteers in consent document review and revising templates to highlight key aspects of a trial while relocating less interesting information to a supplement (e.g., data protection). These approaches are necessary to improve understanding without compromising ethical standards.

## LIMITATIONS

The analyses of 900 screening failures were based on statistically dependent observations as the same participant may occasionally have participated in screening visits multiple times. This is common in study centers, as they often have a participant database and contact volunteers known to the study center. It was not possible to identify from the dataset which measurements came from the same subject, so information about the covariance structure was lost. Therefore, no correlation analysis was performed. Furthermore, the majority of the retrospectively analyzed trials were investigator‐initiated trials; therefore, the broad applicability to commercial contract research facilities may be limited.

The five interviews were conducted with HVs from a single trial site. Although most participants had previously taken part in clinical trials at other trial sites, this was not systematically assessed. Therefore, a selection bias cannot be excluded.

Due to the trial set‐up and protection of employees of the trial sites, the HV responses to the distributed questionnaire had to be anonymized. Therefore, it was not possible to perform the questionnaire analyses stratified by individual trial centers. Potential site‐related effects, such as differences in recruitment practices or HV populations, could therefore not be assessed.

## PRACTICAL IMPLICATIONS

In summary, practical implications arise from our study: We strongly encourage a revision of HV eligibility criteria, on‐site quality assessment, including assessment of recruitment strategy, and public education addressing misconceptions regarding risk and compensation. This may reduce stigma and support sustainable recruitment.

## FUNDING

No external funding was received for this work.

## CONFLICT OF INTEREST

The authors declared no competing interests for this work.

## AUTHOR CONTRIBUTIONS

E.K., M.K., and A.B. wrote the manuscript. E.K., M.K., C.V., V.S.W., and A.B. designed the research. E.K., M.K., M.S., S.B., R.B., F.M., and A.B. performed the research. M.B. contributed new analytical tools. E.K., M.K., N.S., and A.B. analyzed the data.

## PREVIOUS PRESENTATION OF WORK

This work was presented as poster at the 10^th^ German Pharm‐Tox Summit, March 25–28, 2025, Hannover and the 11^th^ German Pharm‐Tox Summit, March 17–20, 2026, Düsseldorf.

## Supporting information


Data S1.
