# Supplementary material for: How to Optimize Healthy Volunteer Screening and Enrolment in Clinical Trials: A Qualitative and Quantitative Approach to Evaluate Processes and Healthy Volunteers' Perceptions
Source: Clin Pharmacol Ther. 2026 Sep 9:10.1002/cpt.70462. Online ahead of print. doi: 10.1002/cpt.70462 (PMC13555743; doi:10.1002/cpt.70462)
Supplement: Supplementary file 1 [file CPT-9999-0-s001.pdf]

Supplemental material to

How to optimize healthy volunteer screening and enrolment in clinical trials: a qualitative and quantitative approach to evaluate processes and healthy volunteers' perceptions

Authors and affiliations

Evelyn Krohmer[1], Manuela König[1], Cathrin J. Vogt[1], Marina Streckebein[2], Sybille Baumann[3], Max Blumenstock[4], Ruwen Böhm[5], Fabian Müller[6,7], Nadja Struß[1], Viktoria S. Wurmbach[1], Antje Blank[1]

1: Internal Medicine IX - Department of Clinical Pharmacology and Pharmacoepidemiology, Heidelberg University Hospital, Im Neuenheimer Feld 410, 69120 Heidelberg, Germany; 2: CRS hVIVO, Mannheim, Germany; 3: CRS, Berlin, Germany; 4: Insitute of Medical Informatics, Heidelberg University, Heidelberg, Germany; 5: Socratec, Erfurt, Germany; 6: Boehringer Ingelheim Pharma GmbH & Co. KG, Biberach an der Riß, Germany; 7: Institute of Experimental and Clinical Pharmacology and Toxicology, Friedrich-Alexander-Universität Erlangen-Nürnberg, Erlangen, Germany

Corresponding author

Dr. Evelyn Krohmer

Heidelberg University

Medical Faculty Heidelberg / Heidelberg University Hospital

Internal Medicine IX - Department of Clinical Pharmacology and Pharmacoepidemiology

Im Neuenheimer Feld 410

69120 Heidelberg, Germany

Email: EvelynNatalie.Krohmer@med.uni-heidelberg.de

Keywords

Healthy volunteers, Phase I clinical trials, screening failure, motivation

Supplementary tables

Table S1: Code definitions of interview main categories

**Main category**	**Definition**
Positive experiences with study participation	Includes reports on past participation in clinical drug trials that are considered positive
Negative experiences with study participation	Includes reports of past participation in clinical drug trials that are considered negative
Factors influencing the decision for/against study participation	Reports on the decision-making process regarding participation in a study and aspects/framework conditions of a study that positively or negatively influence this decision
Risk perception	Reports on specific aspects/framework conditions of a hypothetical study that the test subject associates with a high or low risk or cites as general reasons for a possible perception of risk.
Perception of the informed-consent meeting	Reports on how the interviewee perceived the informational discussion or how they felt before/during/after the informed-consent meeting
Expectations for the informed-consent meeting	Statements about the interviewee’s expectations before entering into the informed-consent meeting
Reactions of the social environment to participation in the study	Reports on reactions from the social environment of the person interviewed (friends, family, etc.) to their participation in the study
Recruitment method	Reports on the recruitment method(s) that led to contact with the study center.
Duration of the decision-making process	Reports on the duration of the decision-making process regarding study participation
Determination of financial compensation	Reports on assumptions and hypotheses regarding how the expense allowance for clinical drug trials is determined

Table S2: Structure and item distribution of the study questionnaire

Domain	*Items*				*Total*
	Mandatory single-choice	Mandatory multiple-choice	Conditional	Optional open	
Recruitment	6	1	-	1	*8*
Informed consent	5	-	-	-	*5*
Motivation	5	-	-	1	*6*
Decision process regarding trial participation	55	-	-	3	*58*
Public perception of trials	9	-	2	-	*11*
Total	80	1	2	5	*88*

Table S3: Characteristics of included studies in the retrospective analysis

**Characteristic**	**Total [n]**	**Percentage [%]**
**Study type**	30	
**First in human**	3	10
**Pharmacodynamic**	1	3.3
**Pharmacokinetics: Bioequivalence**	1	3.3
**Pharmacokinetics: DDI**	13	43
**Pharmacokinetics: Other**	12	40
**IIT or SIT**	30	
**IIT**	26	87
**SIT**	4	13

DDI: Drug-drug interaction, IIT: Investigator-initiated trial, SIT: sponsor-initiated trial

Table S4: Socio-demographic characteristics of 900 screened healthy volunteers

**Characteristic**	**Category**	**Total [n] (n = 900)**	**Percent [%]**
**Age [y]**		26[a]	18-63[a]
**BMI [kg/m²]**		24[b]	3.68[b]
**Ethnic background**	White/Caucasian	711	79
	Not given/Other	141	16
	Asian	18	2.0
	More than one Ethnicity	17	1.9
	Hispanic or Latino	12	1.3
	Black or African American	1	0.1
**Occupation**	Unknown	431	48
	Student	340	38
	Employed	119	13
	Unemployed	10	1.1
**Prior trial participation**	No participation in any trials	353	41
	Previous participation in trials	507	59
	Unknown	40	-
**Prior participation in a clinical trial[c]**	Non-clinical trial	83	16
	Clinical trial	234	46
	No further information given	190	37
**Sex**	Male	454	50.4
	Female	442	49.1
	Unknown	4	0.45

BMI: body mass index; Missing data may be the case, when the screening process was prematurely ended due to identification of exclusion criteria. For 4 participants source data sheets were not available [a]: Age is reported as median with range; [b]: BMI is reported as mean and standard deviation. [C]: This assessment includes only HVs who reported to previously participated in trials. Due to rounding, percentages may not add up to 100%.

Table S5: Reasons and outcomes for screening follow-up

Reasons for screening follow-up	Total [n]	Percentage [%]
**Blood biochemistry**	76	73.1
**Blood routine**	12	11.5
**Vital signs**	7	6.7
**Urinalysis**	7	6.7
**ECG evaluation**	2	1.9
**Total**	104	100
**Included after follow-up**	61	58.7

Table S6: Reasons and outcomes for re-screening

Reasons for re-screening	Total [n]	Percentage [%]
**Organizational reasons**	9	37.5
**Blood routine**	3	12.5
**Blood biochemistry**	3	12.5
**Study specific reasons**	3	12.5
**Other**	2	8.3
**Vital signs**	1	4.2
**ECG evaluation**	1	4.2
**Physical examination**	1	4.2
**Use of medication that could interfere with IMP**	1	4.2
**Total**	24	100
**Included after re-screening**	19	79.2

Supplementary figures

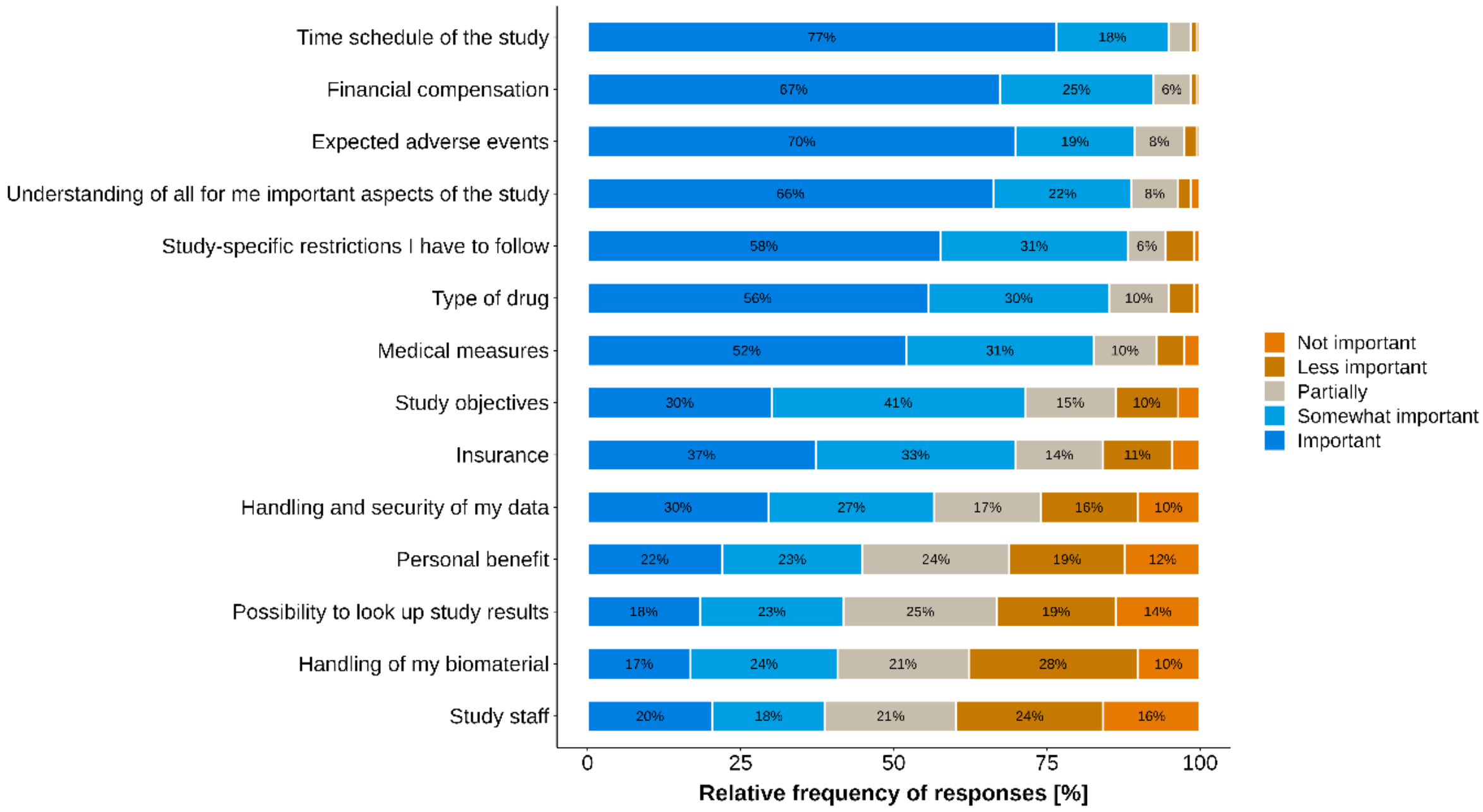

Figure S1: Rated importance of information presented during informed consent. Percentage values >5% are displayed in the corresponding bar.

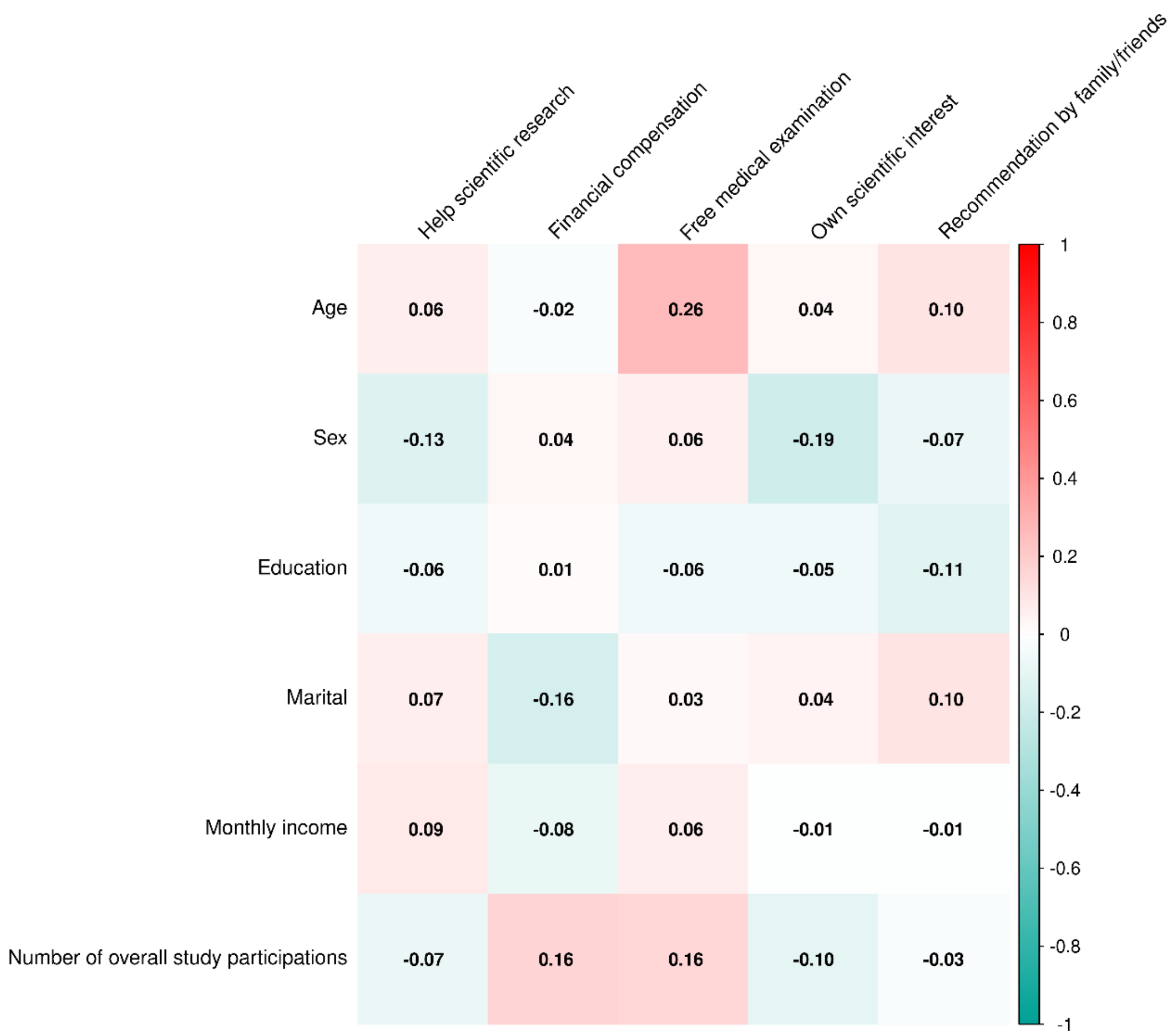

Figure S2: Correlation matrix of population variables and main motivation for trial participation. A correlation of 0.3 or greater would have been deemed significant. No significant correlation was observed.

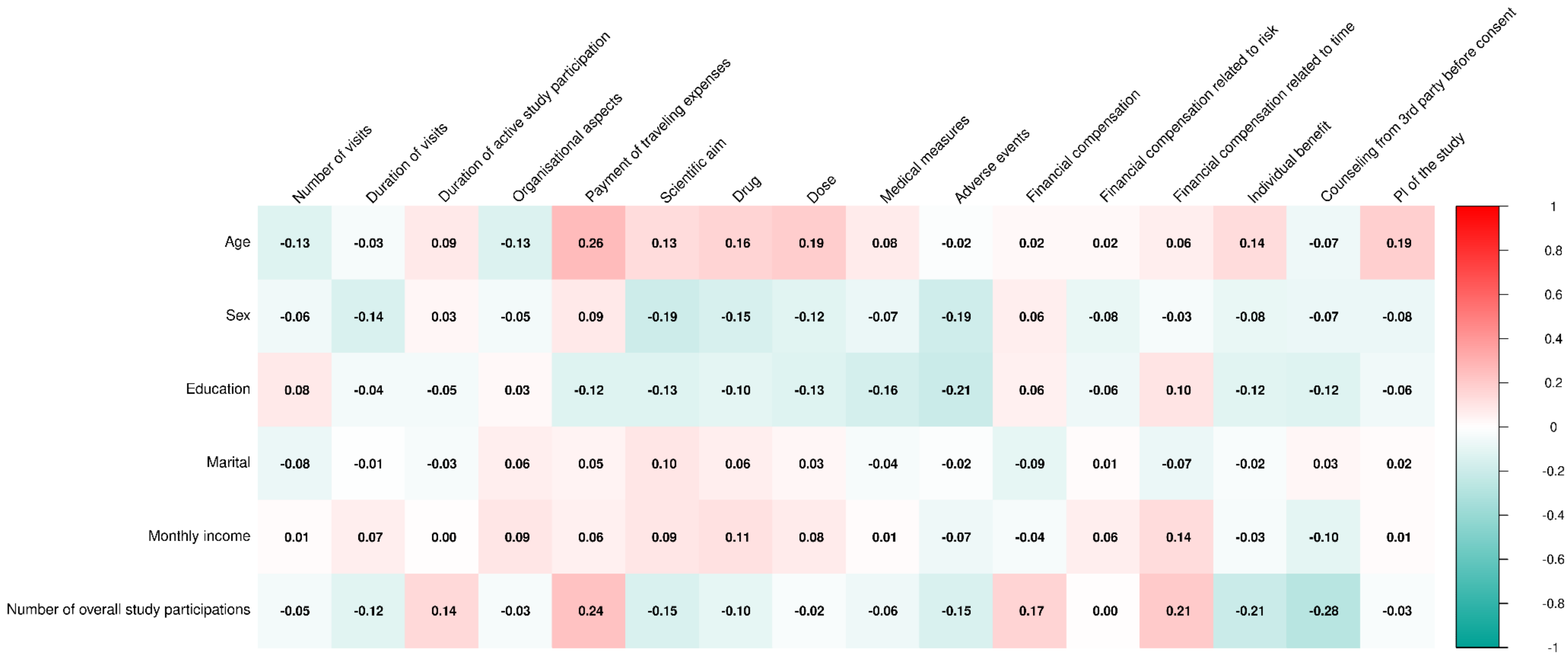

Figure S3: Correlation matrix of population variables and the importance rating of different aspects for trial participation. A correlation of 0.3 or greater would have been deemed significant. No significant correlation was observed.
